# Usefulness of multiplex PCR methods and respiratory viruses’ distribution in children below 15 years old according to age, seasons and clinical units in France: A 3 years retrospective study

**DOI:** 10.1371/journal.pone.0172809

**Published:** 2017-02-24

**Authors:** Benoit Visseaux, Gilles Collin, Houria Ichou, Charlotte Charpentier, Samia Bendhafer, Madalina Dumitrescu, Lahcene Allal, Bogdan Cojocaru, Luc Desfrère, Diane Descamps, Laurent Mandelbrot, Nadhira Houhou-Fidouh

**Affiliations:** 1 IAME, UMR 1137, INSERM, Université Paris Diderot, Sorbonne Paris Cité, Laboratoire de Virologie, Hôpital Bichat, AP-HP, Paris, France; 2 Service de Néonatologie, Hôpital Louis Mourier, AP-HP, Colombes, France; 3 Service des Urgences pédiatrique, Hôpital Louis Mourier, AP-HP, Colombes, France; 4 Service de Maternité, Hôpital Bichat, AP-HP, Paris, France; 5 Service de Maternité, Hôpital Louis Mourier, AP-HP, Colombes, France; Kliniken der Stadt Köln gGmbH, GERMANY

## Abstract

**Background:**

To date, only influenza and RSV testing are recommended for respiratory viruses’ detection in paediatric units. In this study, we described, according to seasons, ages and clinical units, the results obtained in children (<15 years old) by multiplex-PCR (mPCR) tests allowing a quick and wide range detection of all respiratory viruses. These results were also compared with RSV specific detection.

**Methods:**

All nasopharyngeal mPCR and RSV tests requested by clinicians in our French teaching hospitals group between 2011 and 2014 were retrospectively included. All repeated samples for the same children in the same month were discarded.

**Results:**

Of the 381 mPCR tests (344 children) performed, 51.4% were positive. Positivity and viral co-infection rates were higher in the 6–36 months old strata (81% and 25%, *p*<0.0001 and *p =* 0.04, respectively). Viral distribution showed strong variations across ages. During specific influenza epidemic periods, only 1/39 (2.5%) mPCR tests were positive for influenza and 19/39 (48.7%) for other viruses. During specific RSV epidemic periods, only 8/46 (17.4%) mPCR tests were positive for RSV and 14/46 (30.4%) for other viruses. 477/1529 (31.2%) of RSV immunochromatography-tests were positive. Among the negatives immunochromatography-test also explored by mPCR, 28/62 (31%) were positive for other respiratory viruses.

**Conclusion:**

This study provides a wide description of respiratory viruses’ distribution among children in hospital settings using mPCR over 3 years. It emphasizes the number of undiagnosed respiratory viruses according to the current diagnosis practice in France and gives a better picture of respiratory viruses identified in hospital settings by mPCR all over the year in France.

## Introduction

Acute respiratory tract infections (ARTIs) are a major worldwide cause of morbidity and mortality in childhood. Respiratory viruses are the most common cause of such infections and the introduction of molecular detection techniques has considerably improved the detection of these pathogens. Indeed, new multiplex PCR tests now allow a fast detection of a wide range of respiratory viruses [1] including recently described viruses as human metapneumovirus [2], human bocavirus [3] and coronavirus NL63 [4] or HKU1 [5]. Molecular tests also changed the understanding of some older respiratory viruses group such as for rhinovirus, commonly associated only with the common cold and now described as providing severe ARTIs in young children [6–8]. Despite these new knowledges only a few respiratory viruses, such as RSV or metapneumovirus, have been well established as highly pathogenic [9,10]. The causative role of other respiratory viruses is still discussed, mainly as they can be also detected in asymptomatic patients, especially during childhood [11–14].

PCR tests use is largely increasing for respiratory virus’s detection but is still limited by their high cost. Several works studied the cost-effectiveness of rapid multiplex PCR (m-PCR) testing in emergency department and demonstrated a slight cost-effectiveness of such approaches [15,16]. Despite these few studies, international recommendations are still unclear about respiratory viruses testing and their use in clinical care. Thus, the American Association of Paediatrics do not recommend to use any respiratory viruses test [17,18]. The Paediatric Infectious Disease Society and the Infectious Diseases Society of America recommend to use sensitive and specific tests for rapid respiratory viruses’ detection in children above three months of age with community acquired pneumonia [19]. Yet, it does not provide recommendation about younger children. Moreover, stopping antibiotic therapies is recommended for influenza viruses but the recommendations do not statute about other respiratory viruses. Finally, the Royal College of Paediatrics and Child Health and European Society of Paediatric Infectious Diseases states the increasing availability of PCR viral testing, but does not provide any recommendation about their use or management of positive results [20]. This lack of recommendation is explained by the few number of cost-effectiveness studies and the physician’s reluctance to reduce the antibiotic use and other investigations (urine and blood testing) when a virus is detected [16]. Thus, the use of multiplex PCR tests and their benefits for respiratory viruses’ detection in clinical settings still need to be reinforced. In France, to our knowledge, only two studies using mPCR have been conducted in children. Both of these studies were conducted during a single winter season. The first study was conducted in Caen teaching hospital during the particular winter season 2009–2010 and demonstrated the high level of non-RSV and non-flu viruses during this period [21]. The second study was conducted in Toulouse teaching hospital during the winter season 2010–2011 in children attending the emergency unit [22]. These both studies demonstrated a high level of non-RSV non-flu viruses (53 and 46%, respectively) during; or the year after, the particular influenza A H1N1v2009 pandemic. These observations may be useful to be verified over a larger period of time, with a comparison to the adults’ population during the same period of time and especially since the re-emergence of influenza A H3N2 strains that represent the majority of influenza A circulating after the H1N1v2009 pandemic [23,24].

This retrospective study was conducted over a large three-year period, from 2011 to 2014, in a French teaching hospitals group. We describe the distribution of respiratory viruses using mPCR, across age strata, clinical units and seasons.

## Patients and methods

### Patients and period

All nasopharyngeal samples performed in children (<15 years) from the 1^st^ May 2011 to the 30^th^ April 2014 in two hospitals (Louis Mourier and Bichat-Claude Bernard hospitals) and tested on clinician’s request for respiratory viruses or specific RSV detection were retrospectively included. According to local guidelines [25], mPCR were used for children admitted in hospital for respiratory infection. All repeated samples, defined as samples issued from a same patient in the same month, were excluded. Adult population (> 15 years) was recruited in the same conditions for the four adult hospitals of our hospital group (Beaujon, Bichat Claude Bernard, Bretonneau and Adelaïde Hautval hospitals). All our hospitals are located in Northern Paris area.

In accordance with French ethical rules for epidemiological surveillance studies and the rules in place at APHP (Assistance Publique des Hôpitaux de Paris), an oral consent from the patient or parent was collected at admission in hospital and before the anonymous collection of data obtained in routine care. As all the anonymous data were collected and analyzed on site, no specific application to regulatory authorities was required.

### Multiplex PCR tests

During the study period, three m-PCR tests were used. The Respifinder^®^ 19 (Pathofinder^®^, Maastricht, Netherlands) was replaced by the A Respifinder^®^ 22 (Pathofinder^®^, Maastricht, Netherlands) [26] in January 2014 and the Filmarray Respiratory Panel (BioFire Diagnostics, Salt Lake City, USA) [27] was used on physician request from June 2102 to the end of the study. All these tests were not able to differentiate rhinovirus and enterovirus. Thus, they were grouped as picornavirus (rhinovirus + enterovirus) for our analysis. Respifinder^®^ 19 and FilmArray^™^ tests, representing 51.2% of all included tests, were not able to detect bocaviruses. All these tests showed comparable performances in the literature [26,28,29] as well as in our internal comparisons and methods validations. Their reliability was assessed during all the study period using European quality control (QCMD, Glasgow, UK).

### RSV immunochromatographic test

All RSV immunochromatographic (RSV-IC) detection were performed for children >36 months old and during the RSV epidemic periods (from September to March), using the Alere BinaxNOW^®^ RSV immunochromatographic kit (Alere Scarborough, Scarborough, USA).

### Statistical analysis

The distribution of viral findings was analysed according to the month of sampling, age strata and the use of RSV-IC or m-PCR. Patients were stratified into several age groups: 0–6 months-old, 6–36 months-old and 3–15 years-old. Results from the 0–6 months-old were also analysed according to the prescribing units divided in two categories: neonatology unit and other paediatrics’ units. Comparisons between groups were performed using exact Fisher tests with the R software v3.2.2. All tests were performed with a type I error of 0.05.

## Results

### Multiplex PCR results

**Fig 1:**
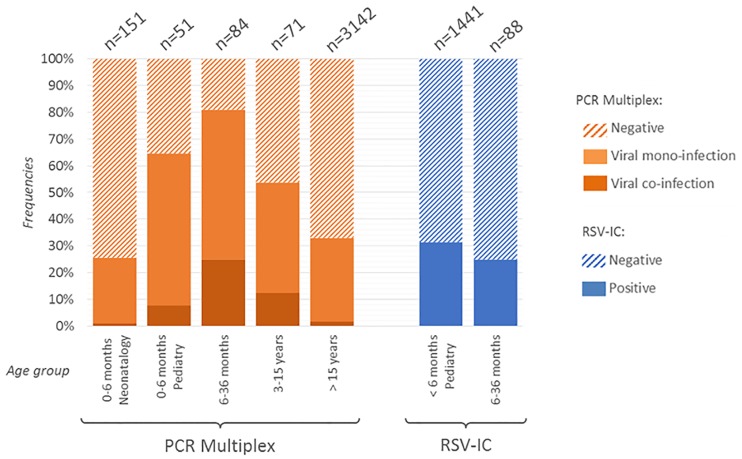
Positivity rates obtained by multiplex PCR and RSV specific immunchromatographic tests according to age and medical units.

**Fig 2:**
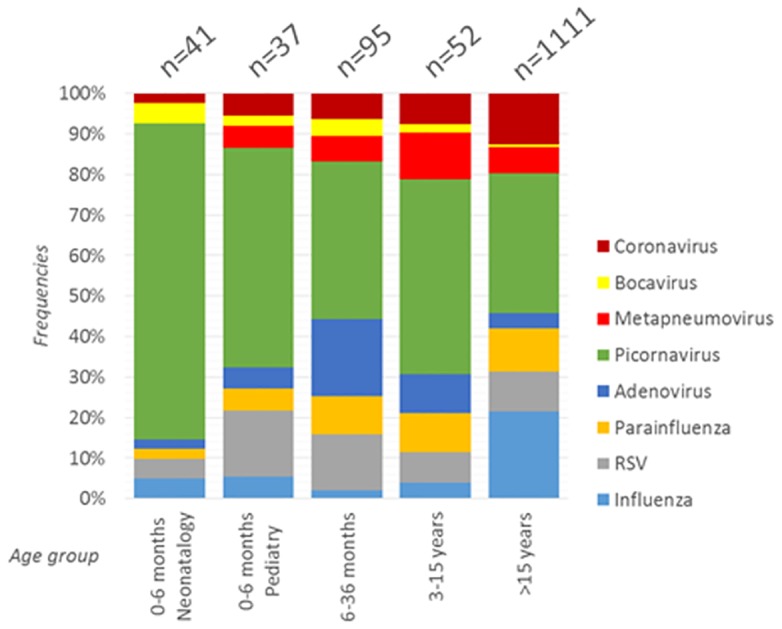
Distribution of all respiratory viruses isolated across age strata. The total number of isolated viruses is indicated at the top of each bar.

During the study period, 381 mPCR tests were performed among 344 children. According to our definition of repeated samples, children cannot be included a second time in the 30-day period following the first sample. The median interval between two inclusion for a same child (n = 37) was 76 days (Inter-Quartile Range [IQR] = 52–168). The children median age at inclusion was 3.2 months [IQR = 0.8–23.7], 48.8% were female. Global positivity rate was 51.4% (196 samples), 9.2% (35 samples) corresponded to viral co-infections. Frequencies of positive and viral co-infection results across age groups are depicted in Fig 1. The identified viruses were picornavirus (n = 125, 52.7%), RSV (26, 11.0%), adenovirus (26, 11.0%), parainfluenza (17, 7.2%), metapneumovirus (14, 5.9%), coronavirus (13, 5.5%), influenza (8, 3.4%) and bocavirus (8, 5.2% of all identified viruses with Respifinder^®^ 22 only able to detect this virus group). In comparison, 3142 adults’ samples were included during the same period among 2103 patients. The median delay between two inclusions for a same patient (n = 1039 samples) was 106 days [IQR = 57–243]. No patient was included a second time if there is less than 30 days since the previous sample. The adult population showed a lower positivity rate (51.4 *vs* 33.1%, *p* = 0.009) and a lower viral co-infection rate (9.2 *vs* 1.9%, *p*<0.001). Viral distribution was statistically different between adults and children (*p*<0.001) (Fig 2). Three virus groups were more represented in children: adenovirus (11 *vs* 3.9%, *p*<0.001), picornavirus (52.7 *vs* 34.4%; *p*<0.001) and bocavirus (3.4 *vs* 0.6%; *p* = 0.002). On the contrary, two viruses’ groups were less represented among children: influenza (3.4 vs 21.5%; *p*<0.001) and coronavirus (5.5 *vs* 12.6%; *p* = 0.002). RSV, metapneumovirus and parainfluenza groups presented similar prevalence in both populations.

**Fig 3:**
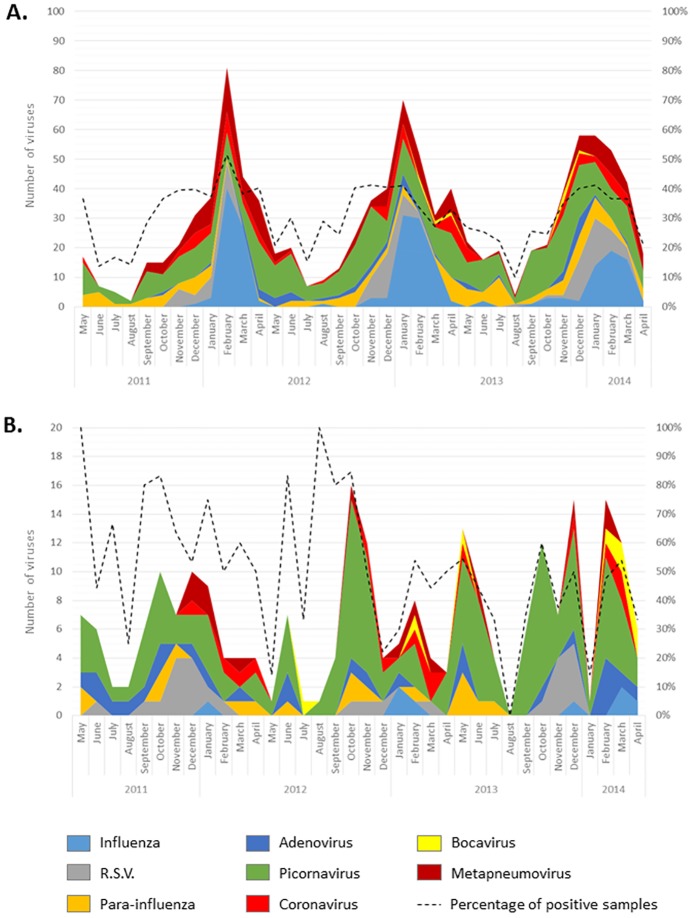
Temporal distribution of respiratory viruses identified by multiplex PCR. (A) in adults (<15 years) and (B) in children (>15 years).

Several differences appear for viruses’ distribution among children in paediatric units and adults. For example, RSV had a tendency to be more frequent in children < 36 months than in all patients > 36 months old (16.8 vs 10.6%, *p*<0.10), as classically described. More interestingly, adenoviruses were more frequent in children between 6 and 36 months old than in all other patients (23.3 vs 4.3%, *p*<0.0001). Influenza was also very low for all children strata but highly frequent among adults (3.4 vs 21.5%, *p*<0.0001). Some temporal variations between children and adults can also be observed during the three years of this study (Fig 3). Thus, from May to October 2011, the adenovirus group was largely present in children but absent in adults. Inversely, parainfluenza viruses were absent in children from September 2013 to April 2014, despite an active circulation among > 15 years old patients.

Neonatology and paediatric unit showed slightly similar positivity and co-infection rates as well as virus distribution. Thus, among the 151 children <6 months old in neonatology unit, global positivity and viral co-infection were 24.5 and 1.3%, respectively. In comparison, among the 51 children of the same age group in paediatric unit, global positivity and co-infection were statistically higher 56.9 and 7.8%, respectively (*p*<0.001). The distribution of viral groups between these units presented some differences that did not reach a significant level (*p* = 0.3). The most frequent viral group was picornavirus in both populations but was more represented in neonatology than in paediatric units (78.0 vs 54.1%) (Fig 1 and Table 1). This higher representation of picornavirus group may be explained by the asymptomatic carriage of such viruses among adults in neonatology units.

**Table 1:** Characteristics of patients and multiplex PCR results across units and age strata.

Characteristics	0–6 mo *(Neonatalogy)*	0–6 mo *(Pediatry)*	*p*	0–6 mo *(Pediatry)*	6–36 mo	3–15 y	*p*	All children	adults	*p*
n samples	151	51		51	84	71		381	3142	
n children	146	51		51	76	66		344	2103	
Sex (% female)	50.6	37.3	*0*.*2*	37.3	39.3	59.2	*0*.02*	48.8	39.3	*<0*.001*
Median age [IQR]	0.9 months [0.4–1.9]	1.7 months [0.8–4.1]	*<0*.001*	1.7 months [0.8–4.1]	16.1 months [9.2–25.3]	54.6 months [44.9–147.6]	-	3.2 months [0.8–23.7]	59.1 years [47.9–67.8]	-

* Statistically significant value with a type I error of 0.05

Regarding mPCR results across children age strata in paediatric units, positivity rates were statistically different with the highest rates observed in the 6–36 months group: 81.0% and 25.0% for positivity and coinfection rates, respectively. Main children characteristics are depicted in Table 1. Despite some variations, the viral distribution was not statistically different across age groups. Yet, RSV presented a slightly decreasing prevalence among older children with prevalence at 16.2% of all identified viruses in the 0–6 months old strata to 13.7 and 7.7% in the 6–36 months and 3–15 years strata, respectively. The adenovirus group had a slight tendency to be more frequently identified in the 6–36 months group (18.9%) compared to the 0–6 months and 3–15 years groups (5.4 and 9.6%, respectively, *p* = 0.09).

In order to estimate how many diagnoses would have been missed by the use of a specific RSV or influenza detection during their epidemic periods, we analysed separately all the results obtained during the active circulation of each of these virus according to the data obtained through the French RENAL hospital surveillance network for influenza and RSV [30]. During their respective epidemic periods, RSV and influenza always encountered for 10 to 40% and 10 to 50% of all viruses identified in our laboratory, respectively. During RSV epidemic periods 63 children were sampled in paediatric units. 18/63 (28.6%) were positive for RSV, including 3 viral coinfections (2 with picornavirus and 1 with metapneumovirus), and 30/60 (50.0%) were positive for other respiratory viruses. Among the latter, 38 viruses have been identified: 21 picornaviruses, 5 coronaviruses, 4 adenoviruses, 3 influenza viruses, 3 metapneumoviruses, 1 parainfluenza and 1 bocavirus. During the influenza epidemic periods, 58 children were sampled in paediatric units. 4/58 (6.9%) were positive for influenza, including one coinfection with a picornavirus, and 34/58 (58.6%) were positive for other respiratory viruses. Among the latter, 46 viruses have been identified: 17 picornaviruses, 8 metapneumoviruses, 7 coronaviruses, 7 adenoviruses, 3 bocaviruses, 2 RSV and 2 parainfluenza.

The proportion of each virus group identified in viral co-infections was statistically different between virus groups (*p* = 0.02, Table 2), ranging from 19.2% for RSV to 65.4% for adenovirus. Two virus groups presented a statistically lower level of viral co-infections compared to all other viral groups: RSV (19.2%, *p* = 0.04) and picornaviruses (20.8%, *p*<0.0001). On the contrary, only adenovirus presented a statistically higher proportion of viruses identified among such coinfections than in other viral groups (*p* = 0.02). No specific viral association emerged and all respiratory viruses seem able to cohabit with any other virus.

**Table 2:** Proportion of each respiratory viruses detected by mPCR in viral mono or co-infection.

	Influenza	RSV	Parainfluenza	Adenovirus	Picornavirus	Metapneumo-virus	Bocavirus	Coronavirus
Number identified in mono-infection	4	21	6	9	99	8	3	8
Number identified in co-infection	4	5	11	17	26	6	5	5
*% of viruses found in co-infection*	*50*.*0%*	*19*.*2%*	*64*.*7%*	*65*.*4%*	*20*.*8%*	*42*.*9%*	*62*.*5%*	*38*.*5%*

### RSV immunochromatography results

As currently recommended [25], these tests have been used during the French RSV epidemic periods in children below 36 months old. Overall, 1529 RSV-IC samples were performed from 1508 children, 477 (31.3%) were positive with a slight decrease in the 6–36 months strata (31.6% vs 25.0% for 0–6 and 6–36 months strata, respectively, *p* = 0.24) (Fig 1). Interestingly, the reverse pattern is observed with the use of mPCR with a slight increase between the 0–6 and the 6–36 months groups (64.7% and 81.0% of positive viruses detection, respectively, *p* = 0.06).

Among children presenting a negative RSV-IC test, 43 were also tested by mPCR in the same 2 days period. Sixteen (33.3%) were positive by mPCR and 4 displayed viral co-infections. The mPCR test has been done specifically on physician request, reflecting a probable more symptomatic population. Among them, 36 (83.7%), 6 (14.0%) and 1 (2.3%) were belonging to the 0–6, 6–36 and >36 months old, respectively. Interestingly, mPCR were more frequently positive among children in paediatric units (7/11, 63.6%) rather than neonatology (4/25, 16.0%, *p* = 0.007). Among the 7 positive mPCR in paediatric units, 4 corresponded to viral co-infections. The two most frequently identified viruses were picornavirus (n = 12) and parainfluenzae (n = 3). Metapneumovirus, adenovirus, bocavirus and coronavirus were also identified (n = 1 in all cases). Underlying the good sensitivity of RSV-IC in children, only one RSV was identified by mPCR among the 43 RSV-IC negative samples. In this particular case, the mPCR test was performed two days after the RSV-IC test. Thus, we cannot conclude if this discrepancy is explained by a lower sensitivity of RSV-IC test or by the apparition of RSV in this child in these two days interval.

## Discussion

This retrospective study provides an unlighted picture of respiratory viruses’ global epidemiology in children in hospital settings using mPCR methods over a large 3 years period in France. The results confirm some of the previous observations made in two other France area, Toulouse and Caen, and during the influenza A H1N1 v2009 emergence period [21,22]. We also completed these previous observations by the analysis of respiratory viruses outside the winter period and by comparing the main differences observed between children and adults’ populations. We also observed the number of respiratory viruses identified after a negative RSV specific testing. These observations are of importance in order to have a better opinion and view of the diagnosis missed by the current practice in France where only RSV and influenza specific testing are recommended during the epidemic periods and no testing at all outside these periods.

We underlined the huge variations of positivity rate and viral distribution of respiratory viruses among various children age groups. Neonatology units presented lower positivity and viral coinfection rates than paediatric units and interestingly closer to those observed in the adult’s population but with a different distribution of respiratory viruses. Thus, this population presented an overrepresentation of picornavirus among all our studied population. In paediatric units, influenza and RSV were never the majority group of viruses identified in our study in all children age strata. Even during the influenza epidemic periods, influenza represented only 6.9% of all identified viruses in children using mPCR. Concerning RSV, the prevalence reached a peak among the 0–6 month’s age strata but at only 16.2% of all identified viruses using mPCR. Even during the specific RSV epidemic period only 28.6 and 31.3% of children in paediatric units were positive for RSV using mPCR or RSV-IC, respectively. These observations are in line with previous studies in France [21,22]. Moreover, the number of other viruses identified during the same period was high in our work: 46.7% of children tested by mPCR in our study during RSV epidemic period were positive for any other respiratory viruses. This pattern was similar in all our children populations. Even when the clinical symptoms conducted the physician to request a RSV-IC test, only 31.3% were positive and 63.6% of negative RSV-IC were positive with another respiratory viruses when tested by mPCR. Taken together, these results underline that RSV testing is badly suited for respiratory viruses’ detection in all children population and may lead to non-optimal care decision such as unnecessary antibiotic use, laboratory testing, radiologic exams or hospitalisations. The lack of a complete respiratory viruses’ detection may also lead to unnecessary isolation or an underestimation of the nosocomial epidemics.

Our study presents some limitations. This is a retrospective epidemiological study without clinical information or assessment of the impact of mPCR on patients’ management that still need to be assessed by specific prospective studies. Another limitation is the use of different mPCR tests. However, the performances of all these tests are similar in both literature and our local validation processes. Concerning bocaviruses, they were not detected by two of our mPCR kits, representing 51.2% of all tested samples. However, this virus group is rare in France and was only detected in 8 samples (5.2% of all samples tested with Respifinder^®^ 22) comprising 3 bocavirus mono-infections and 5 viral co-infections.

Studies of mPCR cost effectiveness are still needed today to endorse a wider use of mPCR. However, detection of all respiratory viruses already allows to a better understanding of viral epidemics at a local and a global scale. For example, in our study, the comparison of results obtained using mPCR in adults and children populations showed some discrepancies as for parainfluenza viruses absent from children in 2014 but heavily circulating in adults population, or for adenovirus overrepresented in children in 2011 but absent in adults. This last event may be in line with some reports about a deadly adenovirus outbreak in children observed in Asia during the same period of time [31–33], suggesting that this outbreak may also have occurred in Europe with the same strains. Unfortunately, samples have not been stored in our hospital and we are not able to investigate this point. A monitoring in real time of all respiratory viruses, only possible by a larger use of mPCR, may help us to identify and explore more closely these specific epidemics.

In conclusion, the use of mPCR tests has led to huge improvements in our ability to detect respiratory viruses. These tests may be useful in order to decrease antibiotic treatment and unnecessary invasive investigations such as blood culture test or radiological exams. To date, the exact role of mPCR is not identified in national or international guidelines, because a lack of consensus on the management of infected children and the few number of cost-effectiveness studies [16]. However, even with the lack of strong cost-effectiveness studies, these tests are already useful for enhanced isolation and management decisions of children presenting severe symptoms, especially for severe respiratory viruses including influenza and RSV, but also para-influenza or metapneumovirus [12,13,34]. Viral co-infections, frequent among children, may also be more easily detected allowing better cohorting decisions.

In this context, the current study emphasizes the number of undiagnosed respiratory viruses according to the current diagnosis practice in France and gives a better picture of respiratory viruses identified in hospital settings by mPCR all over the year.
